# An Unusual Cardiac Cause of Unilateral Neonatal Wheezing

**DOI:** 10.1155/2019/9638518

**Published:** 2019-04-22

**Authors:** James Brock, Eliezer Nussbaum, Jared Shows, Son Nguyen, Shaun P. Setty

**Affiliations:** ^1^Pediatric Pulmonology Division, Miller Children's Hospital, Long Beach, CA, USA; ^2^University of California, Irvine School of Medicine, Irvine, CA, USA; ^3^MemorialCare Health System, Long Beach, CA, USA; ^4^The Translational Pulmonary and Immunology Research Center, Long Beach, CA, USA; ^5^Pediatric Cardiac Surgery, Memorial Heart & Vascular Institute, Long Beach, CA, USA

## Abstract

A neonate presented three days after birth with left-sided unilateral inspiratory wheezing, intermittent respiratory distress, and desaturations. She was found to have a large ductus arteriosus aneurysm that caused compression of her left mainstem bronchus and left pulmonary artery. This lesion was not identified prior to birth on routine prenatal screening, which included fetal ultrasonography. Diagnosis was made on day of life (DOL) 5 by a computed tomography with angiography scan. On DOL 7, she underwent cardiac surgery which included resection of the ductal aneurysm, patch reconstruction of the transverse aortic arch and descending aorta, patent ductus arteriosus excision, and atrial secundum septal defect repair. There were no postoperative complications, and she has been asymptomatic since.

## 1. Introduction

Aneurysm of the ductus arteriosus is a rare condition that is most commonly found in early infancy, frequently may be associated with complications, and if isolated may undergo spontaneous rapid resolution.

## 2. Case Report

**Figure 1:**
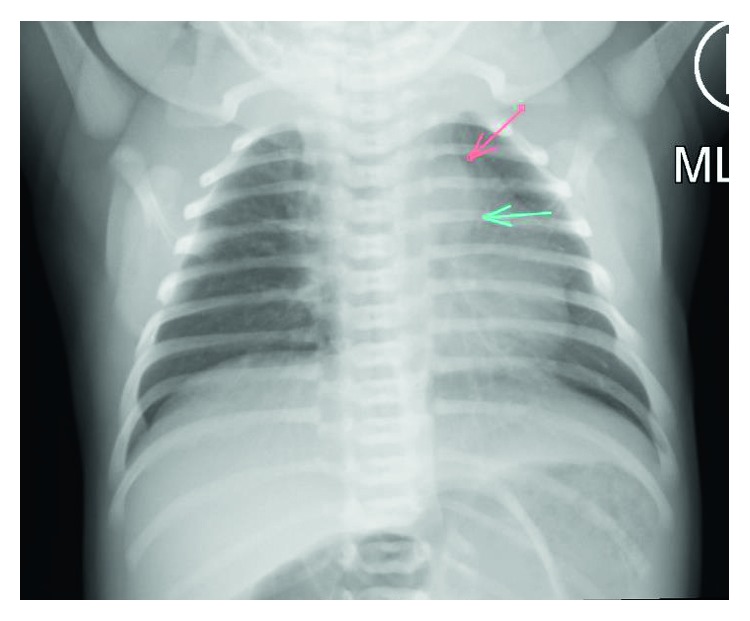
Chest radiograph at presentation: arrows indicate lucency of the ductus arteriosus aneurysm.

A 3.6-kg female born at 41 weeks gestation was noted on day of life (DOL) 3 to have unilateral left-sided wheezing. She had been transferred to the neonatal intensive care unit (NICU) for hyperbilirubinemia requiring phototherapy and subsequently developed respiratory difficulty. In the NICU, her initial chest radiograph (CXR) demonstrated a focal opacity near the medial aspect of her left upper lobe (Figure 1).

**Figure 2:**
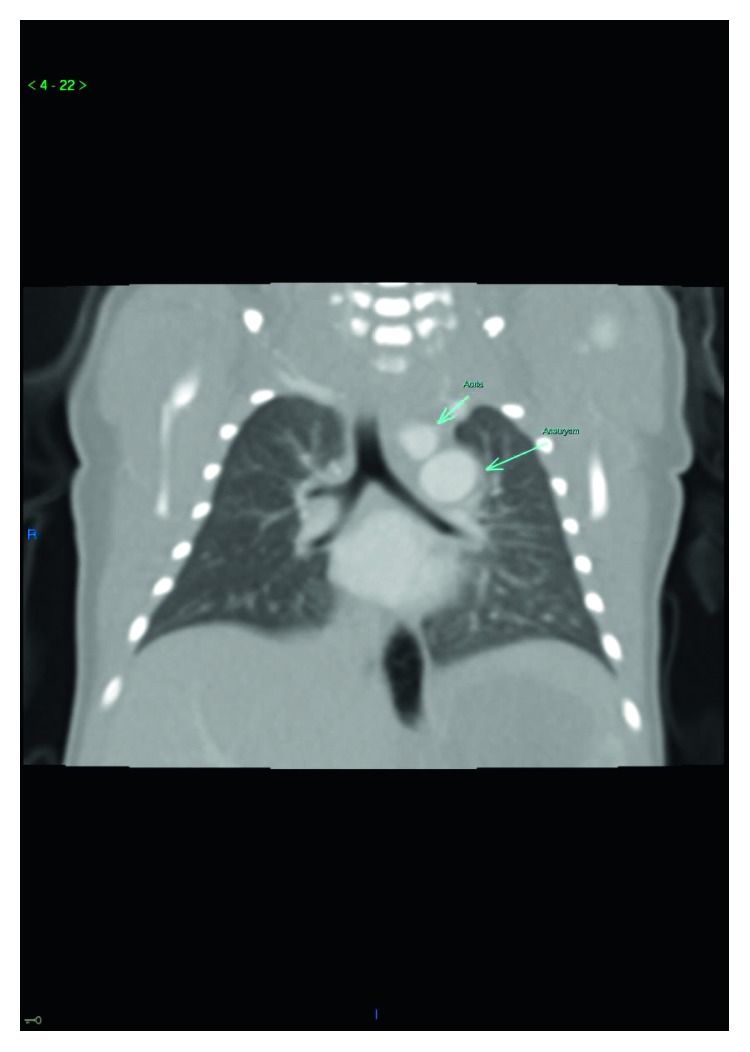
Chest-computed tomography with angiography (CT-A): coronal plane revealing aneurysm relationship to the left bronchus.

**Figure 3:**
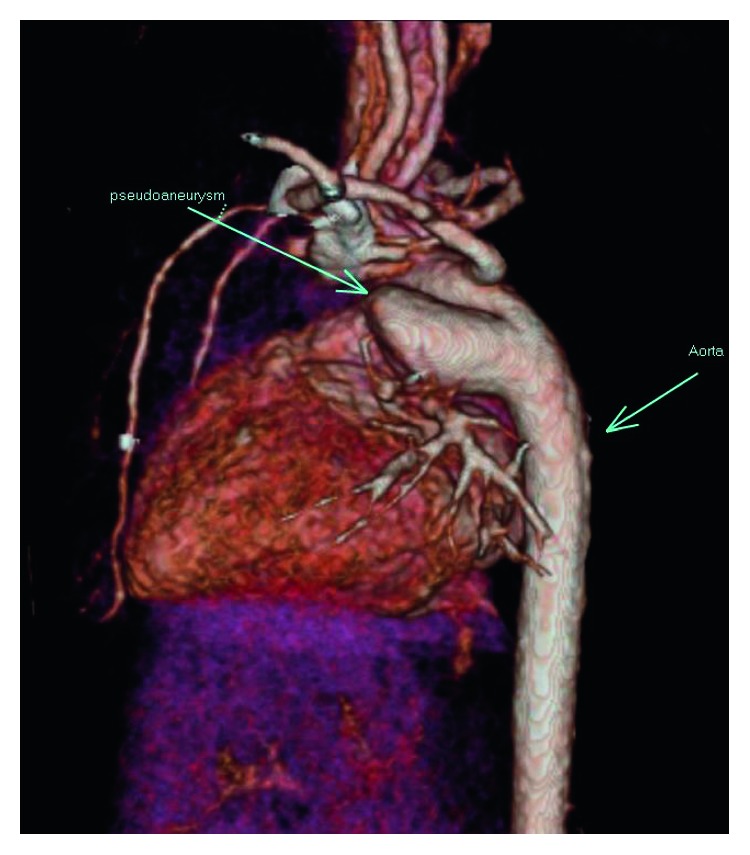
Three-dimensional volume rendering of the thoracic CT-A revealing pseudoaneurysm and relationship with aorta and left pulmonary artery.

Physical examination was notable for intermittent tachypnea with a maximal respiratory rate of 57 breaths per minute and mild subcostal retractions, pulse oximetry of 92% with intermittent desaturations, and inspiratory wheezing noted over her left unilateral chest. On DOL 5, a computed tomography with angiography scan (CT-A) was ordered following a pediatric pulmonology consultation. CT-A revealed a large 17-millimeter ductus arteriosus aneurysm (DAA) (in relation to her 6-millimeter distal transverse aortic arch), which was complicated by an associated large ductal thrombus (Figures 2 and 3). Echocardiogram revealed otherwise normal intracardiac anatomy other than a small 3-4 mm atrial septal defect with left to right shunting, minimal ductal flow detected due to occlusion by thrombus, and left branch pulmonary artery stenosis adjacent to diverticulum. The DAA was not identified prior to birth on routine prenatal screening, which included fetal ultrasonography.

On DOL 7, she underwent cardiac surgery due to the large size of the DAA, compression of adjacent left mainstem bronchus and pulmonary artery, and risk of rupture. The operation included resection of the ductal aneurysm and accompanying abnormal aortic ductal tissue, ligation of patent ductus arteriosus on the pulmonary arterial end, reconstruction of the distal transverse aortic arch, and descending aorta with a large bovine pericardial patch via cardiopulmonary bypass under deep hypothermic circulatory arrest, and upon rewarming, the small secundum atrial septal defect was closed. The DAA was very large in relation to the aortic arch/descending aorta and was very thin walled. Postoperatively, the patient did well and was discharged home on postoperative day 7 with no pulmonary or cardiac issues. Follow-up echocardiography and chest radiography reveal normal findings. She continues to have no respiratory complaints or abnormal clinical exam findings and is thriving.

## 3. Discussion

Unilateral wheezing on physical examination shortly after birth should incorporate imaging workup for anatomical causes, as opposed to assigning the origin of air flow turbulence and airway obstruction as a functional abnormality. Ductus arteriosus aneurysm (DAA) was first described at autopsy in 1827 by Martin-Saint-Ange [1] and is considered a rare condition in infancy. Historically, incidence was estimated in 0.5–1% of neonatal autopsies [2]. As modern perinatal imaging and diagnostic modalities have advanced, more recent studies have suggested higher incidence of 1.5% (by fetal ultrasound) [3] to as high as 8.8% in full-term neonates [4]. Suprasternal and parasternal transthoracic echocardiogram approaches can reveal a triple star sign that comprises the DAA, aortic arch, and pulmonary artery [4]. Isolated DAA is most commonly asymptomatic and typically presents at less than 2 months [4–6]. The ductus arteriosus is derived from the left sixth aortic arch embryonically and likely to emerge in the third trimester [5]. Proposed mechanisms of DAA pathogenesis include delayed closure of the aortic end of the ductus arteriosus (DA), weakened DA wall due to cytologic necrosis and mucoid degeneration of the media layer of the ductus, or abnormal elastin fibers as seen in connective tissue disorders [7]. Risk factors for DAA include uncontrolled maternal gestational diabetes, large-for-gestational age fetuses, and an association to connective tissue disorders [5–8]. The complication rate of DAA has been reported as high as 30% and includes aneurysm rupture (risk of rupture elevates with increasing size of DAA), embolism, erosion, pulmonary infection secondary to bronchial obstruction, left pulmonary arterial stenosis, thrombosis of the aortic arch or pulmonary arteries, compression of the nearby phrenic, and recurrent laryngeal nerves causing stridor and/or respiratory distress [6, 9, 10]. For asymptomatic cases without evidence of enlargement, observation for spontaneous resolution has been recommended [11, 12]. However, in the setting of symptomatic findings, surgical treatment is advocated [13, 14]. While DAA resection has been shown to be an effective surgical strategy, a recent case demonstrated ligation and decompression to be an effective technique in the absence of rupture or thrombus [10]. The size of the aneurysm plays a role in the options and urgency of treatment, in which a large expanding thin-walled DAA has a greater propensity for rupture. A lateral thoracotomy can be considered depending on the anatomy but due to the large size of this DAA, large neck on the aortic side, need for extensive patching, and risk of rupture, we chose an anterior approach.

**Figure 4:**
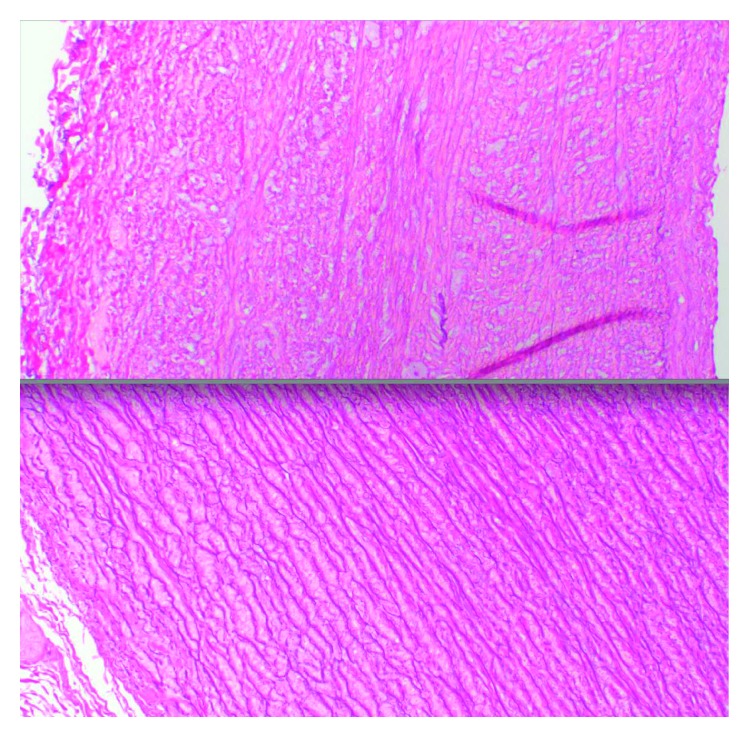
Histopathology: elastic stain 100x (DAA (top) and healthy control (bottom)) revealing tissue disarray.

In this case, the DAA was excised which revealed a significantly large 1.7 × 1.0 centimeter DAA that was nearly completely occluded by thrombus. Our patient was displaying symptoms of respiratory difficulty due to compression of her adjacent airway and vascular structures by the DAA and associated thrombus. In addition to her symptoms, the risk of rupture with the size of the DAA in our opinion warranted urgent surgical repair over conservative therapy as both strategies were discussed with the family. The excised DAA was examined by pathology and showed vascular tissue with myxoid degenerative change and haphazard arrangement of the collagen fibers. Elastic tissue stains were performed and showed incomplete elastic membrane layer, which was compared against normal control tissue (Figure 4).

## 4. Conclusion

Aneurysm of the ductus arteriosus is a rare condition that is most commonly found in early infancy and may undergo spontaneous rapid resolution. While the pathogenesis is not entirely clear, DAA of the neonate occurs congenitally during fetal development and is typically associated with disruption of the elastic components of the ductus arteriosus. In asymptomatic cases diagnosed prenatally or incidentally and which have persisted until birth, conservative management with close monitoring and serial echocardiography until spontaneous closure may be appropriate. However, when associated with symptoms or sequelae of its presence, in addition to a large size, surgical treatment is recommended. Physical findings suggestive of air flow turbulence or airway obstruction shortly after birth should incorporate imaging workup for anatomical causes, rather than to assume the origin to be of a functional abnormality. The findings that can be included in the differential diagnosis are as follows:

- Vascular ring or pulmonary sling
- Tumor (neoplasm vs. benign mass)
- Airway obstruction (e.g., foreign body and mucous plug)
- Congenital heart disease (noncyanotic)
- Congenital pulmonary airway malformation (CPAM)
- Bronchogenic cyst
